# Integrated multi-omics reveals flower color regulatory networks in *Osmanthus fragrans*

**DOI:** 10.1186/s43897-025-00158-y

**Published:** 2026-01-05

**Authors:** Meng Tang, Tao Song, Lydia Pui Ying Lam, Min Zhang, Min Li, Yi-Xue Wu, Fu-Liang Cao, Hong-Guo Chen, Mo-Xian Chen, Ying-Gao Liu, Fu-Yuan Zhu

**Affiliations:** 1 National Key Laboratory for the Development and Utilization of Forest Food Resources, The Southern Modern Forestry Collaborative Innovation Center, State Key Laboratory of Tree Genetics and Breeding, Key Laboratory of State Forestry and Grassland Administration on Subtropical Forest Biodiversity Conservation, College of Life Sciences, Nanjing Forestry University, Nanjing, 210037 Jiangsu Province China; 2 Graduate School of Engineering Science, Akita University, Akita City, 010-8502 Japan; 3 National Forestry and Grassland Administration Engineering Research Center for Osmanthus Fragrans, Hubei University of Science and Technology, Xianning, 437100 China; 4 State Key Laboratory of Crop Biology, College of Life Science, Shandong Agricultural University, Taian, Shandong China; 5 State Key Laboratory of Desert and Oasis Ecology, Xinjiang Institute of Ecology and Geography, Chinese Academy of Sciences, Urumqi, 830011 China

As one of the top ten traditionally famous flowers in China, *Osmanthus fragrans* holds significant cultural and economic value due to its unique flower color and fragrance. Flower color, an essential ornamental characteristic of *O. fragrans*, varies across different cultivars, ranging from white, light yellow, yellow, golden yellow, orange to carmine. Flower color is primarily dependent on the content and composition of carotenoid and anthocyanin pigments (Wang et al. 2018). For flower breeding to generate new colors for ornamental purposes, it is important to understand the underlying mechanism and molecular players that regulate *O. fragrans* flower color. To this end, we conducted SWATH-MS-based quantitative proteomics using five representative *O. fragrans* cultivars with white (cv. Shenxue or SX), light yellow (cv. Yingui or YG), yellow (cv. Jinqiugui or JQG), orange (cv. Dangui or DG), and carmine (cv. YanZhi Hong or YZH) petals (Fig. 1A) (Chen et al. 2021; Ludwig et al. 2018).

**Fig. 1:**
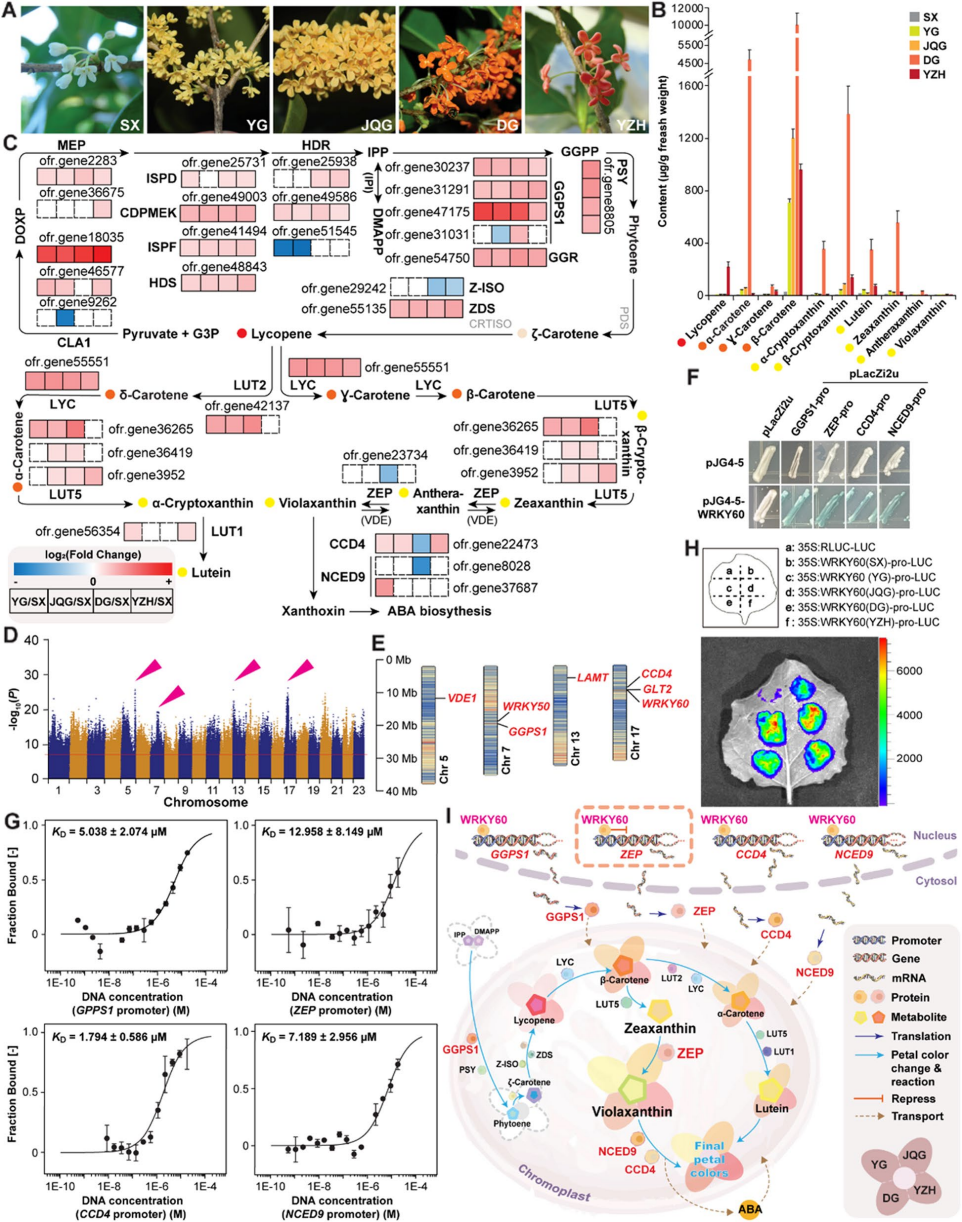
Elucidation of metabolism and regulatory networks of flower color in O. fragrans. **A** Flower colors of O. fragrans cultivars. Flower color of Shenxue (SX) is white, Yingui (YG) is light yellow, Jinqiugui (JQG) is yellow, Dangui (DG) is orange, and YanZhi Hong (YZH) is carmine. SX and YZH are new cultivars, which have passed the certification of new variety registration by the International Cultivar Registration Center for Osmanthus (ICRCO). **B** Quantitation of carotenoids by LC–MS/MS analysis. Color of each carotenoid is indicated. Values refer to means ± standard deviations (*n* = 3). **C** DEPs in carotenoid biosynthetic pathway determined by SWATH-MS-based proteomics. SX was used as a control for comparison. Thirty DEPs in carotenoid biosynthetic pathway were found. Most enzymes in the upstream pathway are up-regulated in all 4 comparisons (YD/SX, JQG/SX, DG/SX, and YZH/SX). In contrast, some downstream enzymes, such as zeaxanthin epoxidase (ZEP), carotenoid cleavage dioxygenase 4 (CCD4), nine-cis-epoxy carotenoid dioxygenase 9 (NCED9), are differentially expressed in these cultivars. G3P, glyceraldehyde 3-phosphate; CAL1, cauliflower 1; DOXP, 1-deoxy-D-xylulose 5-phosphate; MEP, 2-C-methyl-D-erythritol-4-phosphate; ISPD, CDP-ME transferase; CDPMEK, 4-(cytidine 5'-diphospho)−2-C-methyl-D-erythritol kinase; ISPF, 2-C-methyl-D-erythritol 2,4-cyclodiphosphate synthase; HDS, hydroxymethylbutenyl diphosphate synthase; HDR, hydroxymethylbutenyl diphosphate reductase; IPP, isopentenyl diphosphate; IPI, isopentenyl diphosphate isomerase; DMAPP, dimethylpropenyl pyrophosphate acid; GGPS1, geranylgeranyl diphosphate synthase; GGR, geranylgeranyl diphosphate reductase; GGPP, geranylgeranyl diphosphate; GGPPS, GGPP synthase; PSY, carotenoid isomerase; PDS, octahydrolycopene dehydrogenase; Z-ISO, ζ-carotene isomerase; ZDS, ζ-carotene dehydrogenase; CRTISO, carotenoid isomerase; LUT, lutein deficient; LYC, lycopene cyclase. In grey, enzymes not differentially expressed. In brackets, enzymes not identified by proteomics. Color of each carotenoid is indicated. **D** and **E** GWAS analysis of loci associated with flower color of O. fragrans. A total of 1,514 flower color-related SNPs involving 851 genes were identified (**D**). Flower color-related SNP loci on chromosome 5, 7, 13, and 17 are indicated. Candidate genes on chromosome 5, 7, 13, and 17 are indicated (**E**). Chr, chromosome. **F** Binding of WRKY60 on the promoter region of ZEP, CCD4, NCED9, and GGPS1 determined by yeast one-hybrid assay. Interactions were determined based on the blue color produced from X-gal on synthetic defined (SD) medium lacking Trp and Ura. Pro, promoter. **G** Binding affinity between WRKY60 and the promoter region of ZEP, CCD4, NCED9, and GGPS1 determined by microscale thermophoresis. Binding coefficient (KD) is indicated. **H** Analysis of the promoter activity of WRKY60 of different cultivars by effector/reporter-based gene transactivation assays in N. benthamiana leaves. **I** Intrinsic mechanism of flower color regulation by WRKY60. WRKY60 (in pink) regulates the expression of carotenoid biosynthetic genes (in red). The four petals represent YG, JQG, DG, and YZH. The process of color change of the petals represents the process of color formation of these four cultivars

In SWATH-MS-based proteomic analysis, 9457 proteins were identified in all 4 comparisons when using white SX as a control, proteins with fold changes ≥ 2 and ≤ 0.5 (*P* < 0.05) were defined as up-regulated and down-regulated, respectively (Supplemental Fig. 1A, B). Functional classification by GO enrichment revealed that the most significant differentially abundant proteins (DAPs) are involved in various biological activities, including oxidation–reduction process and secondary metabolism. Notably, DAPs in pigment metabolism were detected in all comparisons. Consistently, KEGG pathway enrichment analysis showed enrichment of DAPs in metabolic pathways related to flower color formation, including phenylpropanoid and carotenoid biosynthesis (Supplemental Fig. 2). Eleven DAPs in flavonoid biosynthetic pathway were differentially expressed in different comparisons, but anthocyanidin synthase (ANS), the finial enzyme in anthocyanidin biosynthetic pathway, was consistently down-regulated, limiting anthocyanidin production (Gao et al. 2023). Thirty DAPs in the carotenoid biosynthetic pathway were identified, and were mostly up-regulated as the color deepened (Fig. 1C). Collectively, the composition and content of carotenoids, but not anthocyanidins, determine flower color.

Anthocyanin and carotenoid contents were quantified. Negatable amounts of anthocyanidins were detected and had no correlation with flower color. Carotenoid accumulation varied among cultivars and was associated with flower color (Fig. 1B). Trace amounts of carotenoids were detected in SX, causing its white appearance. Orange pigments such as α-carotene and β-carotene, as well as yellow pigments like zeaxanthin and violaxanthin, were the most abundant in orange DG, and less so in light yellow YG and yellow JQG. Red lycopene content is the highest in red YZH.

Carotenoid biosynthesis primarily begins with isopentenyl diphosphate (IPP) and dimethylpropenyl pyrophosphate acid (DMAPP) from the 2-*C*-methyl-D-erythritol-4-phosphate (MEP) pathway. Subsequent reactions involving geranylgeranyl diphosphate synthase (GGPS1), phytoene dehydrogenase (PDS), ζ-carotene isomerase (Z-ISO), ζ-carotene dehydrogenase (ZDS), and carotenoid isomerase (CRTISO) produce red lycopene. Downstream pathways further generate different carotenoids. Lycopene is converted to α-carotene and β-carotene by lutein deficient 2 (LUT2) and lycopene cyclase (LYC), respectively. Further LUT5-catalyzed reactions generate zeinoxanthin and zeaxanthin. Downstream 9-cis-epoxycarotenoid dioxygenase (NCED9) cyclizes violaxanthin or neoxanthin to produce abscisic acid (ABA) (Kavi Kishor et al. 2022). In YZH, LUT2 and LUT5 exhibited minimal expression, consistent with high lycopene content and low downstream carotenoid accumulation (Fig. 1B). In DG, LUT2, LYC, and LUT5 were highly expressed, whereas carotenoid degradation enzymes zeaxanthin epoxidase (ZEP), carotenoid cleavage dioxygenase 4 (CCD4), and NCED9 were down-regulated, resulting in high contents of carotenoids such as α-carotene, β-carotene, and zeaxanthin, and an orange flower color. Furthermore, 32 DAPs related to RNA processing and 4 WRKY transcription factors were identified by proteomics (Supplemental Table 1). RNA processing, particularly alternative splicing, may regulate flower color post-transcriptionally. Carotenoid biosynthetic genes are alternatively spliced in *O. fragrans* during fruit development, likely contributing to fruit color change (Ma et al. 2023) And it was found that anthocyanin accumulation in the spiny Solanum group because of natural promoter variant and alternative splicing (Wang et al. 2022).

As protein functions are often modulated by post-translational modifications (Lee et al. 2023), especially phosphorylation, we performed phosphoproteomics and detected 1940 phosphorylated proteins, 3324 phosphorylated peptides, and 5420 phosphorylation sites. Those with site confidence ≥ 0.7 were selected. Fold changes ≥ 1.5 or ≤ 1/1.5 (*P* < 0.05) were defined as differential changes (Supplemental Fig. 1C, D). Among all comparisons, although the number of phosphorylated proteins and phosphorylation sites was different, the distribution of phosphorylation site types was similar. S is the major phosphorylation site (> 91%). [SP] and [RxxS] are the predominant motifs. Our data also suggest that CCD4 is phosphorylated for the first time and phosphorylation of CCD4 occurs at amino acid residues 24, 28, 29, 69, and 73, primarily on serine (Ser) and threonine (Thr) residues. In vitro protein kinase assays demonstrated that CCD4 can be phosphorylated (Supplemental Fig. 8). Phosphorylation may regulate protein activity, subcellular localization, interaction, and stability (Olsen et al. 2006). Thus, phosphorylation of CCD4 and other proteins may regulate flower color, but further validation is needed.

Next, we conducted GWAS analysis using 48 light yellow cultivars, 20 orange cultivars and DG as a control to identify single-nucleotide polymorphisms (SNPs) associated with flower color (Fig. 1D, Supplemental Table 2). From chromosome 5, 7, 13, and 17, seven significant association genes including transcription factors *WRKY50* and *WRKY60*, and carotenoid metabolic genes *GGPS1*, *CCD4*, glucose transporter 2 (*GLT2*), violaxanthin de-epoxidase (*VDE*), and loganic acid *O*-methyltransferase (*LAMT*) were identified (Fig. 1E). Interestingly, we identified GGPS1 and CCD4 through an overlap analysis of genes obtained from a weighted gene co-expression network analysis (WGCNA) based on SWATH-MS proteomic data, using flower color as a characteristic, for mutual validation (Supplemental Fig. 3). In previous studies, some of the aforementioned genes were found to be associated with color metabolism. In tomato (*Solanum lycopersicum*), several WRKYs can bind to the promoters of carotenoid biosynthetic genes, involving in color change during fruit ripening (Wang et al. 2017). In melon (*Cucumis melo*), *VDE1* expression is highly correlated with carotenoid content.

We further examined the roles of WRKY60 in carotenoid biosynthesis and flower color determination. Yeast one-hybrid assay and microscale thermophoresis indicated that WRKY60 bound to *GGPS1*, *ZEP*, *CCD4*, and *NCED9* promoters (Fig. 1F, G). Effector–reporter assays were conducted to determine activation/repression activities of WRKY60 on each promoter. The effector construct (35S:WRKY60) was coexpressed with each of the reporter constructs harboring promoter sequence of *GGPS1*, *ZEP*, *CCD4*, and *NCED9* in *Nicotiana benthamiana* leaves. Luciferase (LUC) activity with *ZEP* promoter as an effector was decreased compared to that in control (reporter construct only), indicating that WRKY60 repressed *ZEP* expression while the other three genes were not significantly inhibited by WRKY60 (Supplemental Fig. 4). Taken together, WRKY60 repressed *ZEP* expression, a downstream carotenoid degradation enzyme, by binding to its promoter. Consequently, it may reduce ABA biosynthesis from carotenoids, retaining vibrant flower color.

To understand how WRKY60 regulates flower colors in different cultivars, effector/reporter-based gene transactivation assays in *N. benthamiana* leaves were performed to compare WRKY60’s promoter activity in using promoter sequence of different cultivars, which possess SNPs (Supplemental Fig. 5). LUC activity of YG and JQG’s WRKY60 promoter was higher than that of SX and DG (Fig. 1H, Supplemental Fig. 6, Supplemental Fig. 7), suggesting a stronger binding of other transcription factors. Thus, in light yellow YG and yellow JQG, higher transcription of WRKY60 may cause higher repression of *ZEP* expression and accumulation of zeaxanthin and the upstream precursors, which are yellow pigments (Fig. 1B). Collectively, the variation of WRKY60’s promoter activity in different cultivars influences carotenoid accumulation and flower color (Fig. 1I).

In summary, our comprehensive proteomics and GWAS analysis have identified the metabolism, molecular players, and regulatory networks that determine flower color of *O. fragrans*. We further demonstrated that these approaches can effectively identify molecular players and regulatory networks that determine flower color; WRKY60 repressed *ZEP* expression, and the difference of WRKY60’s promoter activities in different cultivars may regulate carotenoid accumulation and flower color. Collectively, our study has provided useful information for breeding of *O. fragrans* to generate novel flower colors.

## Supplementary Information

Supplementary Material 1.

Supplementary Material 2.

Supplementary Material 3.

Supplementary Material 4.

## Data Availability

The mass spectrometry proteomics data have been deposited to the ProteomeXchange Consortium (https://proteomecentral.proteomexchange.org) via the iProX partner repository with the dataset identifier PXD061154 and PXD061155. The datasets and materials generated or analyzed during this study are available from the corresponding author upon reasonable request.
