# Supplementary material for: Integrated multi-omics reveals flower color regulatory networks in Osmanthus fragrans
Source: Mol Hortic. 2026 Jan 5;6:1. doi: 10.1186/s43897-025-00158-y (PMC12766942; doi:10.1186/s43897-025-00158-y)
Supplement: Supplementary file 1 — Supplementary Material 1. [file 43897_2025_158_MOESM1_ESM.docx]

**Supplemental Data**

**Integrated multi-omics reveals flower color regulatory networks in *Osmanthus fragrans***

Meng Tang ^1, a^, Tao Song ^1, a^, Lydia Pui Ying Lam ^2, a^, Min Zhang ^1, a^, Min Li ^4^, Yi-Xue Wu ^1^,
Fu-Liang Cao ^1^, Hong-Guo Chen ^3,^ *, Mo-Xian Chen ^1,^ *, Ying-Gao Liu ^1,5,^ *, Fu-Yuan Zhu ^1, 5,^ *

^1^ National Key Laboratory for the Development and Utilization of Forest Food Resources, The Southern Modern Forestry Collaborative Innovation Center, State Key Laboratory of Tree Genetics and Breeding, Key Laboratory of State Forestry and Grassland Administration on Subtropical Forest Biodiversity Conservation, College of Life Sciences, Nanjing Forestry University, Nanjing, 210037, China.

^2^ Graduate School of Engineering Science, Akita University, Akita City, Akita, 010-8502, Japan.

^3^ National Forestry and Grassland Administration Engineering Research Center for Osmanthus fragrans, Hubei University of Science and Technology, Xianning 437100, China

^4^ State Key Laboratory of Crop Biology, College of Life Science, Shandong Agricultural University, Taian, Shandong, China.

^5^ State Key Laboratory of Desert and Oasis Ecology, Xinjiang Institute of Ecology and Geography, Chinese Academy of Sciences, Urumqi 830011, China.

^a^ These authors contributed equally to this work.

^*^ To whom correspondence should be addressed
Email: fyzhu@njfu.edu.cn, liuyg@sdau.edu.cn, cmx2009920734@gmail.com, and chhg1969@163.com

**List of Materials**

**Supplemental Figure 1.** Summary of differentially abundant proteins (DAPs) of SWATH-MS-based proteomics and phosphoproteomics.

**Supplemental Figure 2.**  Functional classification of differentially abundant proteins (DAPs) by Gene Ontology (GO) and Kyoto Encyclopedia of Genes and Genomes (KEGG) pathway enrichment analysis.

**Supplemental Figure 3.** Candidate genes related to flower color identified by weighted gene co-expression network analysis (WGCNA).

**Supplemental Figure 4.** The activity of WRKY60 on the expression of *GGPS1*, *ZEP*, *CCD4*, and *NCED9* using effector/reporter-based gene transactivation assays in *N. benthamiana* leaves.

**Supplemental Figure 5.** Multiple sequence alignment of WRKY60 promoter and ZEP promoter sequences of different cultivars.

**Supplemental Figure 6.** Analysis of the promoter activity of WRKY60 of different cultivars by effector/reporter-based gene transactivation assays in *N. benthamiana* leaves.

**Supplemental Figure 7.** qRT-PCR analysis of candidate genes.

**Supplemental Figure 8.** Phosphorylation of CCD4 protein in vivo.

**Supplemental Table 1.** DAPs related to RNA processing in YG/SX, JQG/SX, DG/SX, and YZH/SX comparisons identified by SWATH-MS-based proteomics.

**Supplemental Table 2.** Single-nucleotide polymorphisms (SNPs) identifed by GWAS analysis using 48 light yellow cultivars, 20 orange cultivars, with DG was used as a control.

**Supplemental Table 3.** Primers used in this study.

**Supplemental Table 4.** Information of 68 samples.

**Supplemental Materials and Methods**

**Supplemental References**

**
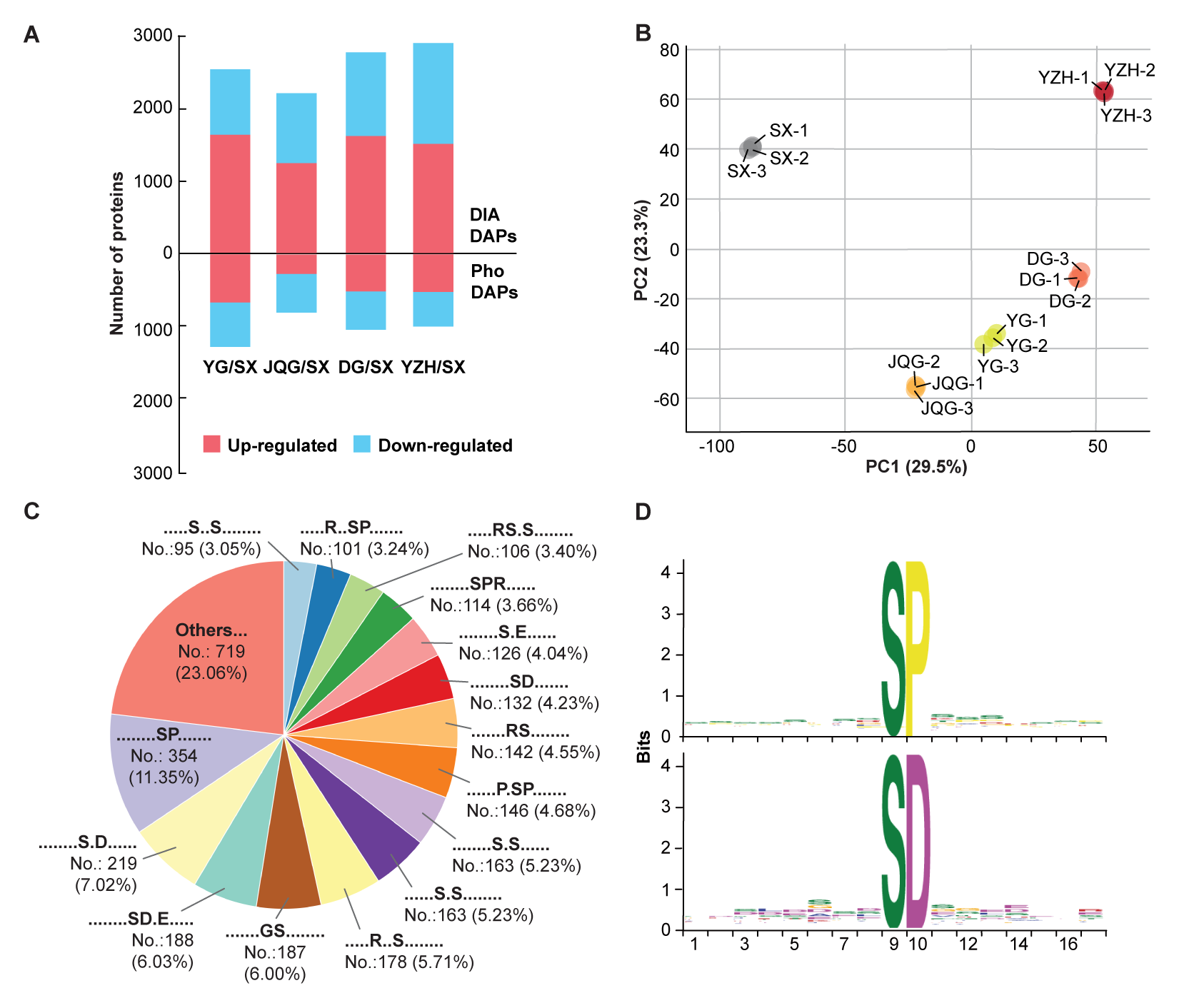
**

**Supplemental Figure 1.** Summary of differentially abundant proteins (DAPs) of SWATH-MS-based proteomics and phosphoproteomics.

**(A)** Number and distribution of DAPs detected in SWATH-MS-based proteomics and phosphoproteomics. DIA DAPs, DAPs identified by SWATH-MS-based proteomics/data-independent acquisition; Pho DAPs, differentially phosphorylated proteins. The white SX petals were served as a control for comparison. Proteins with fold changes ≥2 (*P* < 0.05) were defined as up-regulated proteins (in red), whereas those with fold changes ≤0.5 (*P* < 0.05) were annotated as down-regulated proteins (in blue).

**(B)** Principal component analysis scores plot of DAPs in SX, YG, JQG, DG, and YZH. These five cultivars are separated from each other. Biological replicates of each cultivar are clustered together.

**(C)** Number and distribution of phosphorylation sites detected in phosphoproteomics. The most abundant phosphorylation site was S (more than 91% in all 4 comparisons), followed by T (7-8%) and Y (0-1%). Although the number of phosphorylated proteins and phosphorylation sites were different, the distribution of phosphorylation site types was similar among 4 comparisons, in which S is the predominant site modified by phosphorylation.

**(D)** Protein phosphorylation motifs determined by phosphoproteomics. SX is used as a control for comparison. There are 10, 6, 8, and 9 motifs enriched in YG/SX, JQG/SX, DG/SX, and YZH/SX comparisons, respectively. Among these motifs, [SP], [RxxS], and [SD] are enriched in all comparisons, with [SP] and [RxxS] being the most enriched. [GS] is specifically enriched in YG/SX, whereas [SDD] is specifically enriched in YZH/SX.


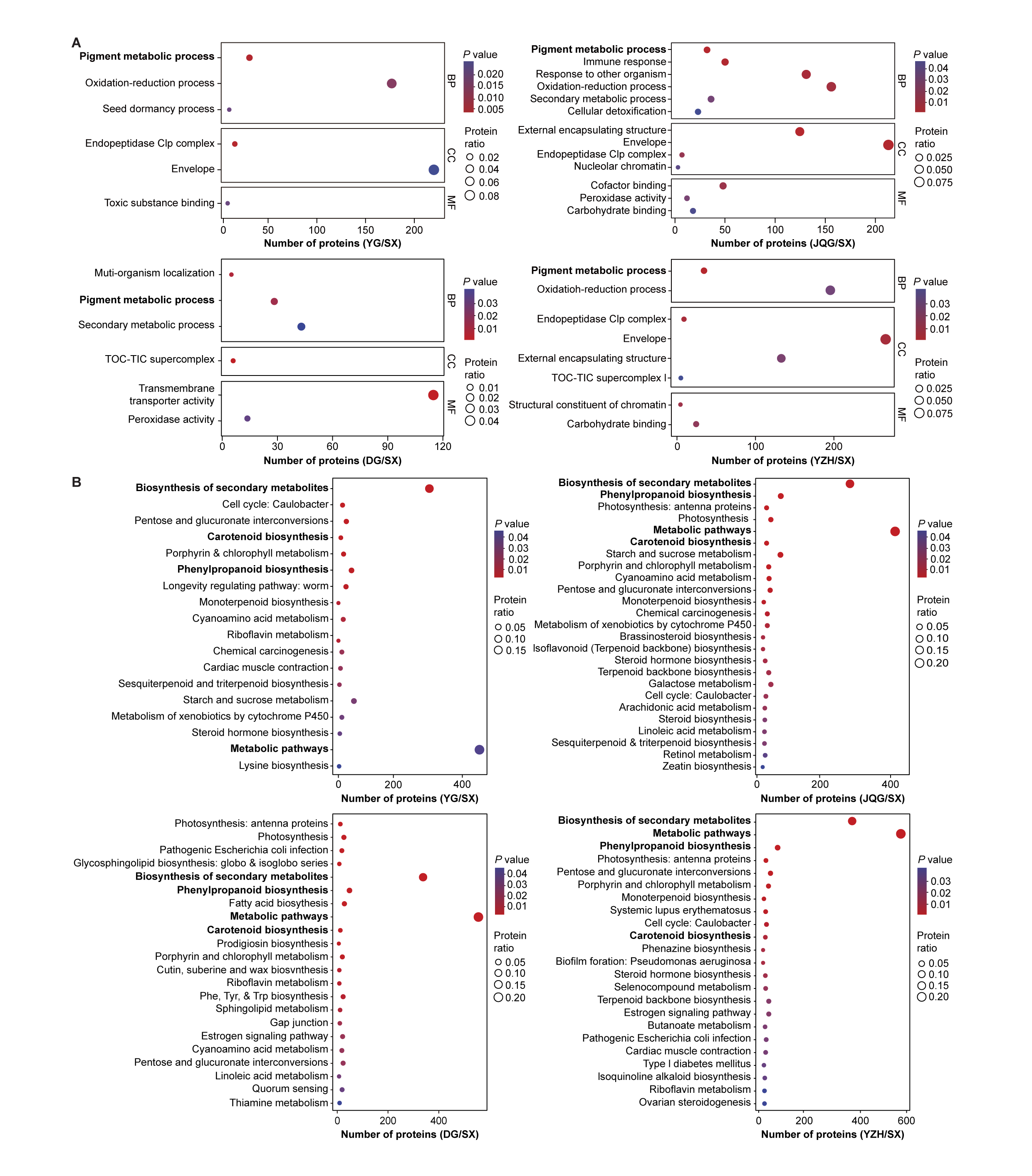


**Supplemental Figure 2.** Functional classification of differentially abundant proteins (DAPs) by Gene Ontology (GO) and Kyoto Encyclopedia of Genes and Genomes (KEGG) pathway enrichment analysis.

**(A)** GO analysis of DAPs of YG/SX, JQG/SX, DG/SX, and YZH/SX comparisons. DAPs in all 4 comparisons are enriched in pigment metabolic process, which are important determining factors of flower color.

BP, biological process; CC, cell component; MF, molecular function.

**(B)** KEGG pathway enrichment analysis of DAPs of YG/SX, JQG/SX, DG/SX, and YZH/SX comparisons. DAPs in all 4 comparisons are enriched in metabolic pathways, including biosynthesis of secondary metabolites, and carotenoid biosynthesis, which are important determining factors of flower color.


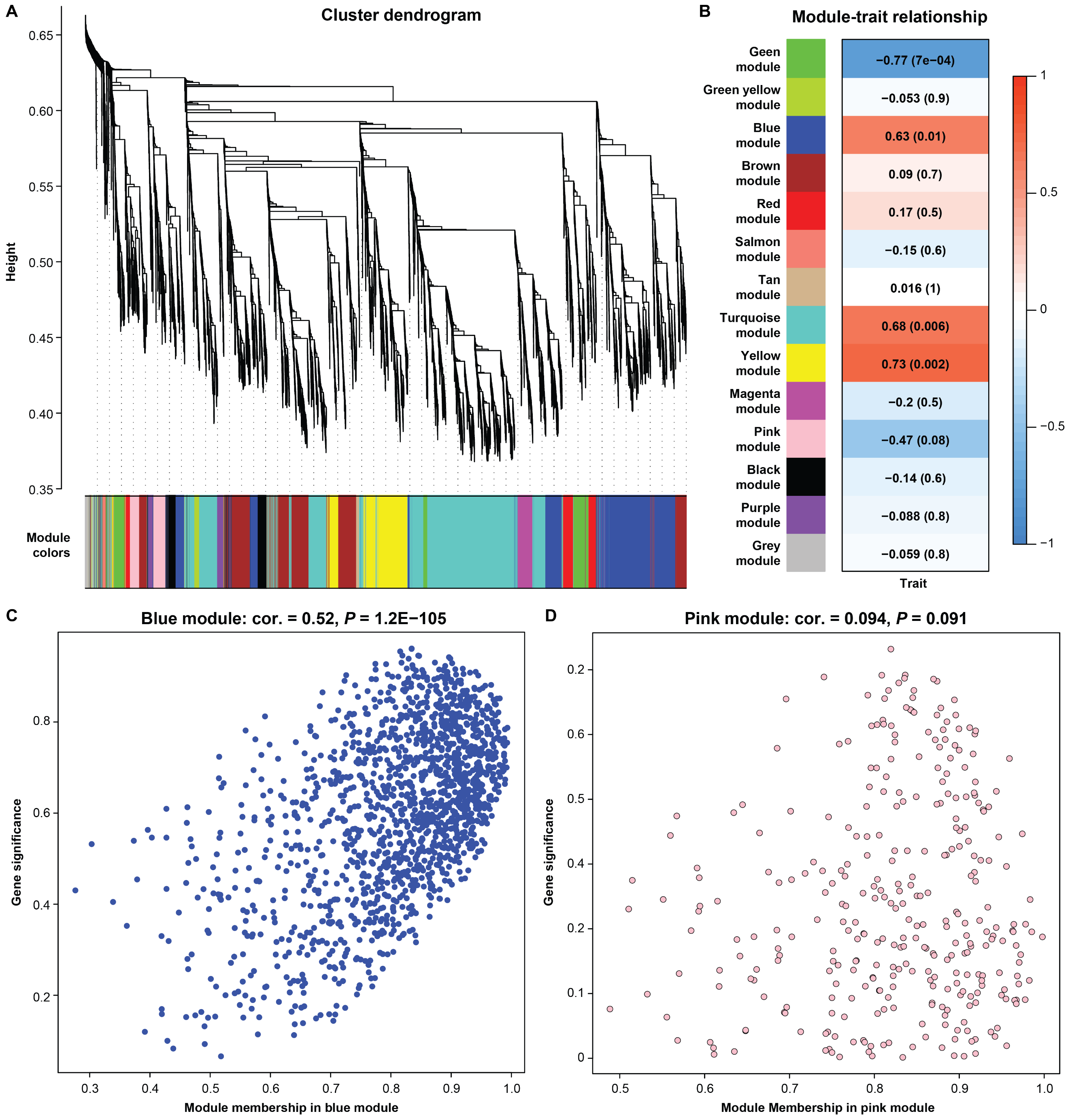


**Supplemental Figure 3.** Candidate genes related to flower color identified by weighted gene co-expression network analysis (WGCNA).

**(A)** Cluster dendrogram of the identified modules. Gene expression was clustered into 14 coexpression modules labelled in different colors and presented in the form of a cluster dendrogram by dynamic splicing method.

**(B)** Correlation between module and trait. Heatmap showing the relationship between modules and traits. Each row represents a module eigengene. The corresponding correlation and *P* value are indicated.

**(C and D)** Relationship between gene significance and module membership in the blue and pink module. There is a highly significant correlation between gene significance and module membership in these modules. *GGPS1* and *CCD4* were identified from blue and pink modules by WGCNA.


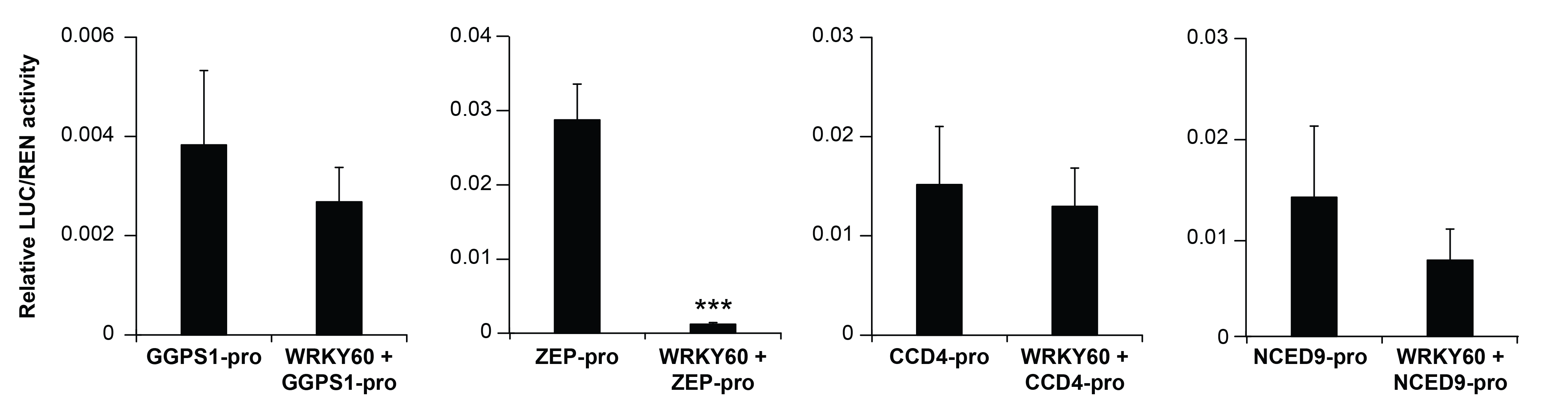


**Supplemental Figure 4.** The activity of WRKY60 on the expression of *GGPS1*, *ZEP*, *CCD4*, and *NCED9* using effector/reporter-based gene transactivation assays in *N. benthamiana* leaves.

WRKY60 repressed *ZEP* expression. Values refer to means ± standard deviations (*n* = 12; ***, *P* < 0.001).

**
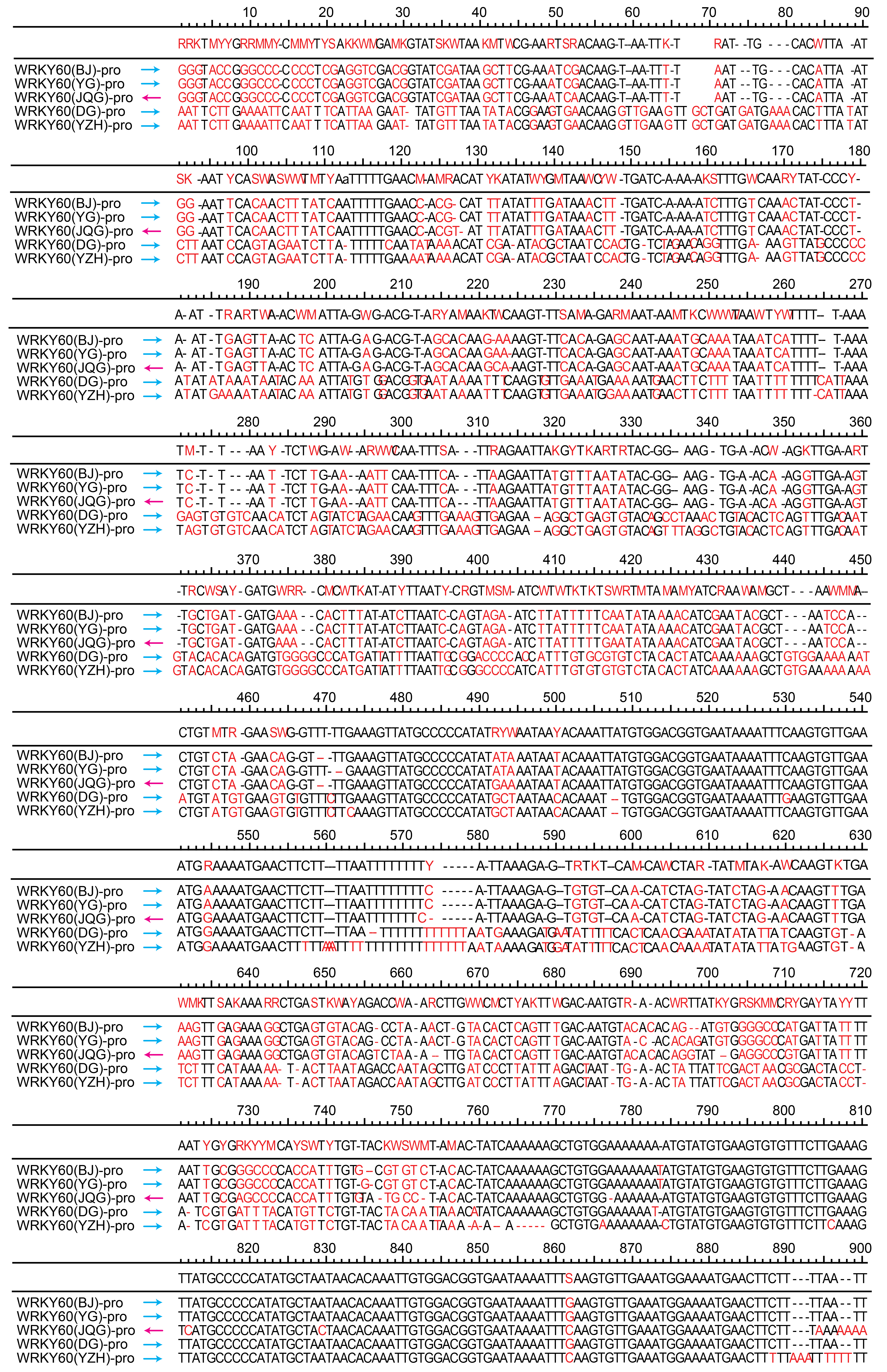
**

**Supplemental Figure 5.** Multiple sequence alignment of WRKY60 promoter and ZEP promoter sequences of different cultivars.

Sequences are from 5ʹ to 3ʹ. In red, variation of DNA sequence.

**
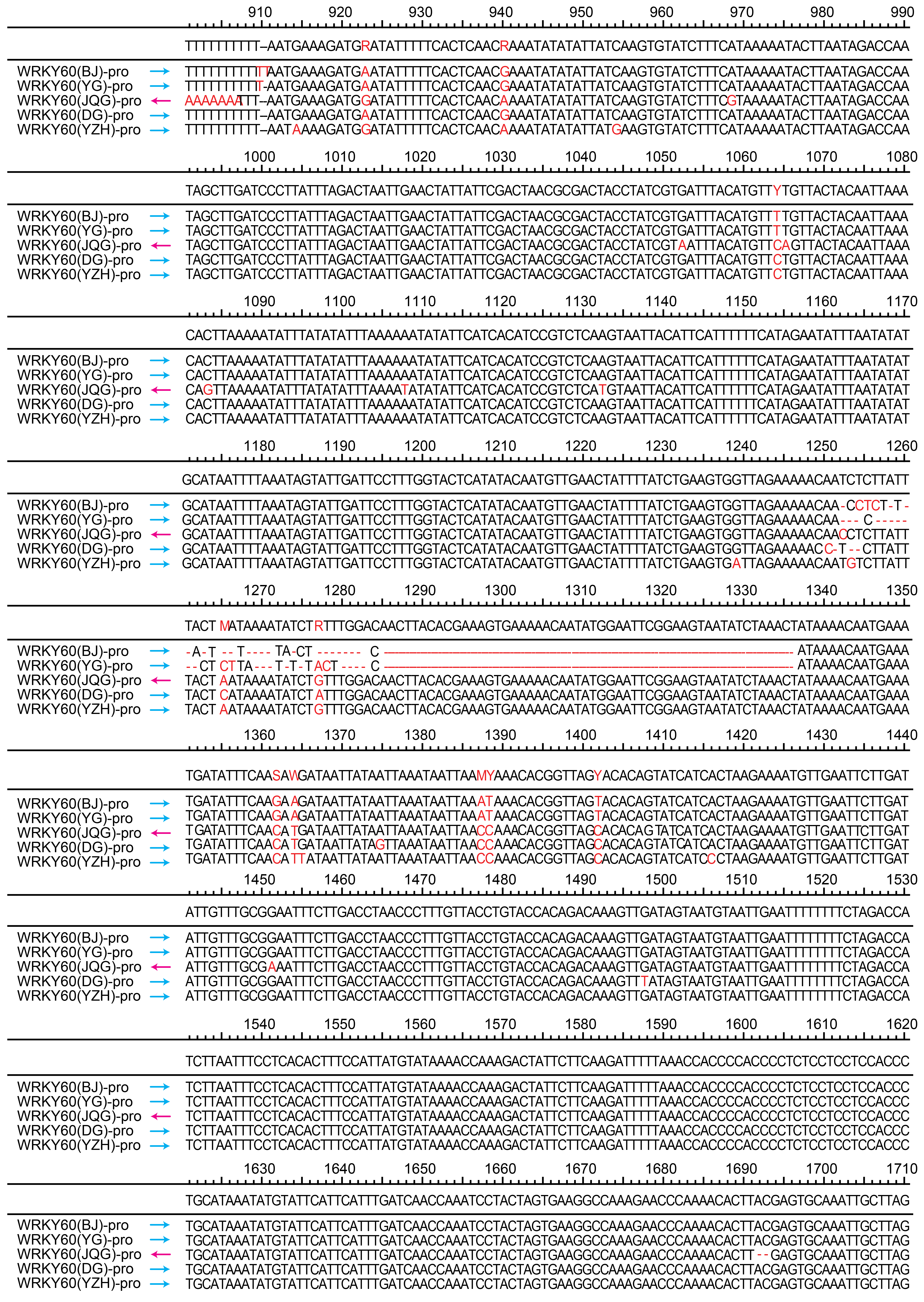
**

**Supplemental Figure 5.** Multiple sequence alignment of WRKY60 promoter and ZEP promoter sequences of different cultivars (continued).

Sequences are from 5ʹ to 3ʹ. In red, variation of DNA sequence.

**
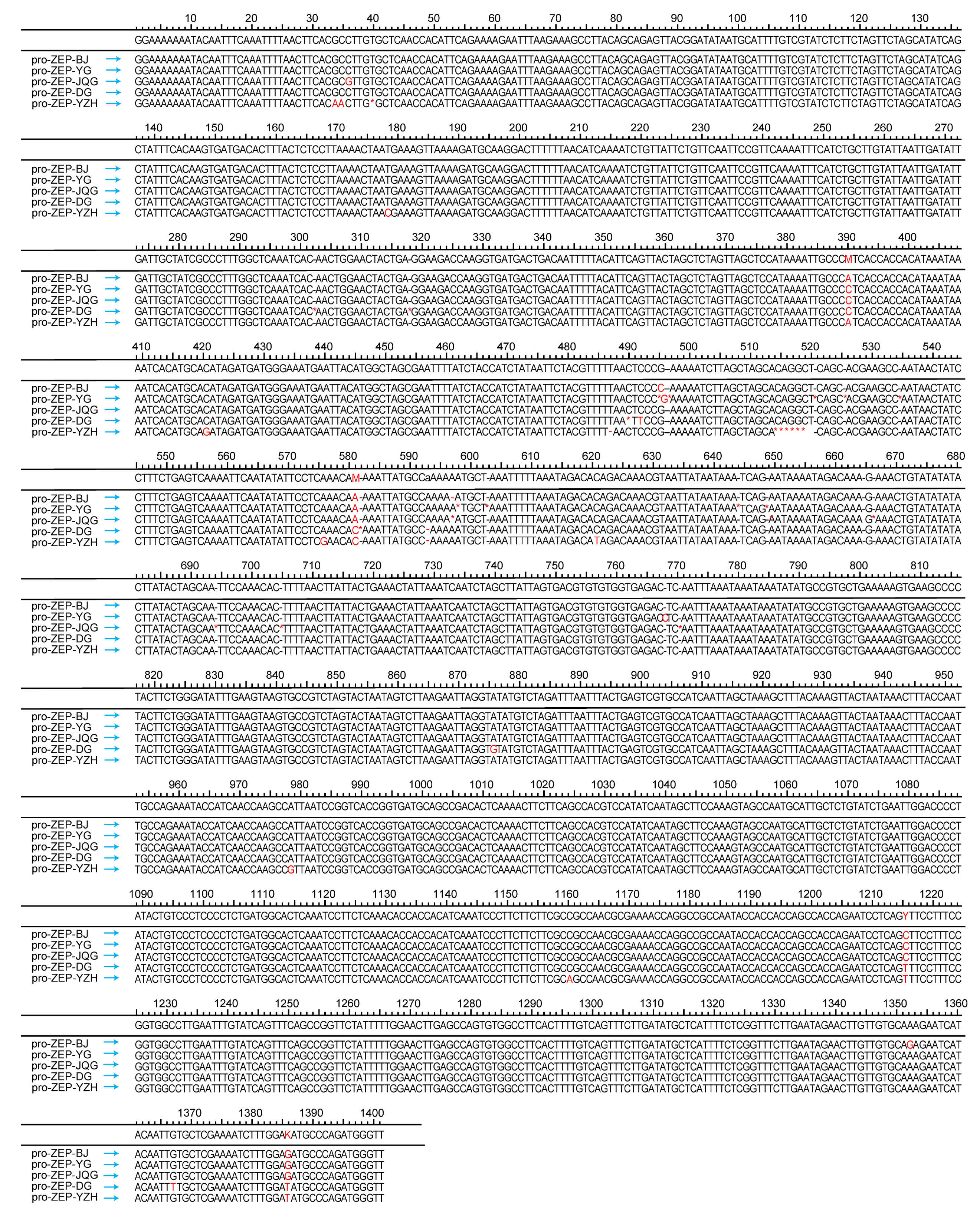
**

**Supplemental Figure 5.** Multiple sequence alignment of *WRKY60* promoter and *ZEP* promoter sequences of different cultivars (continued).

Sequences are from 5ʹ to 3ʹ. In red, variation of DNA sequence.

**
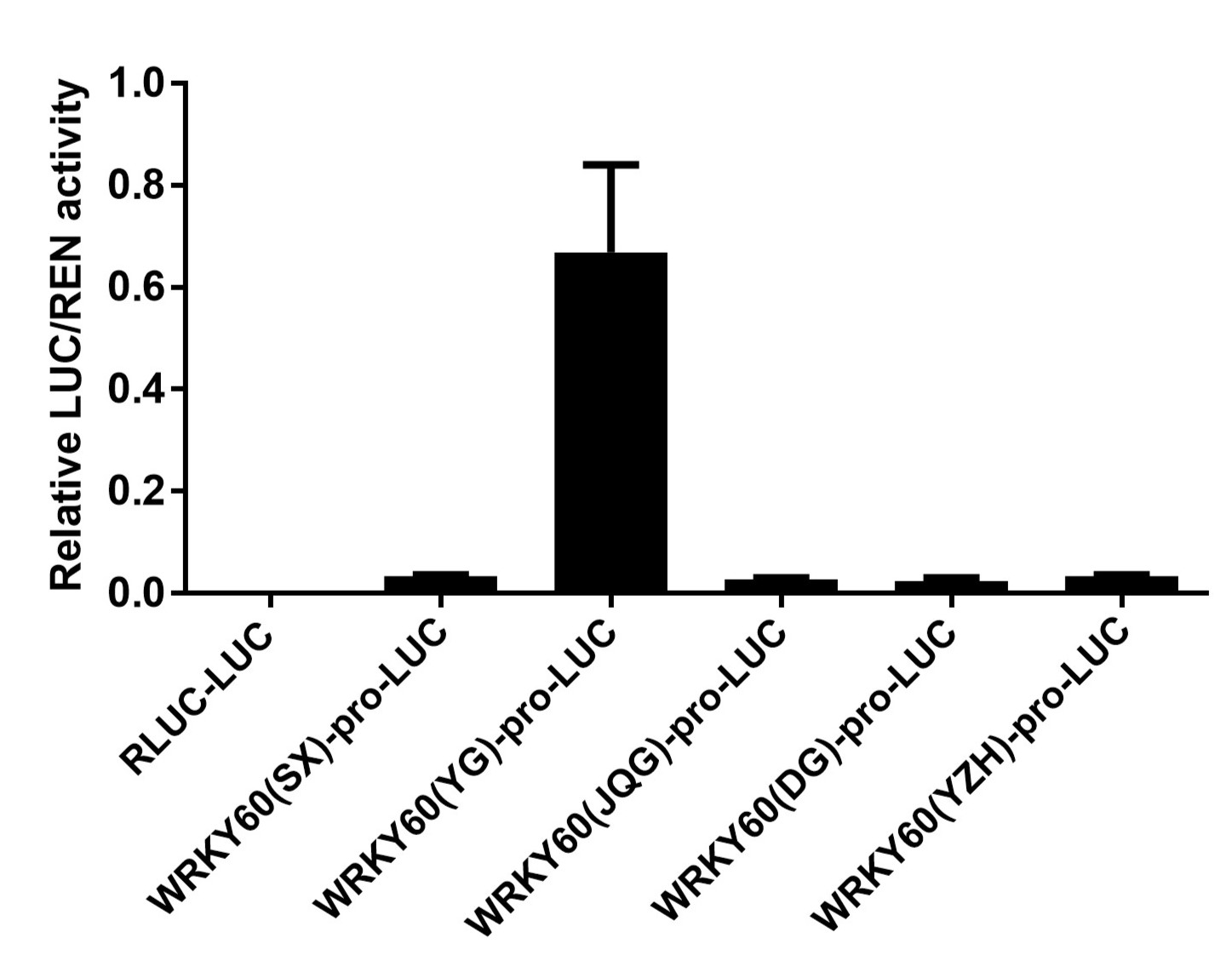
**

**Supplemental Figure 6.** Analysis of the promoter activity of *WRKY60* of different cultivars by effector/reporter-based gene transactivation assays in *N. benthamiana* leaves.

Values refer to means ± standard deviations (*n* = 13).

**
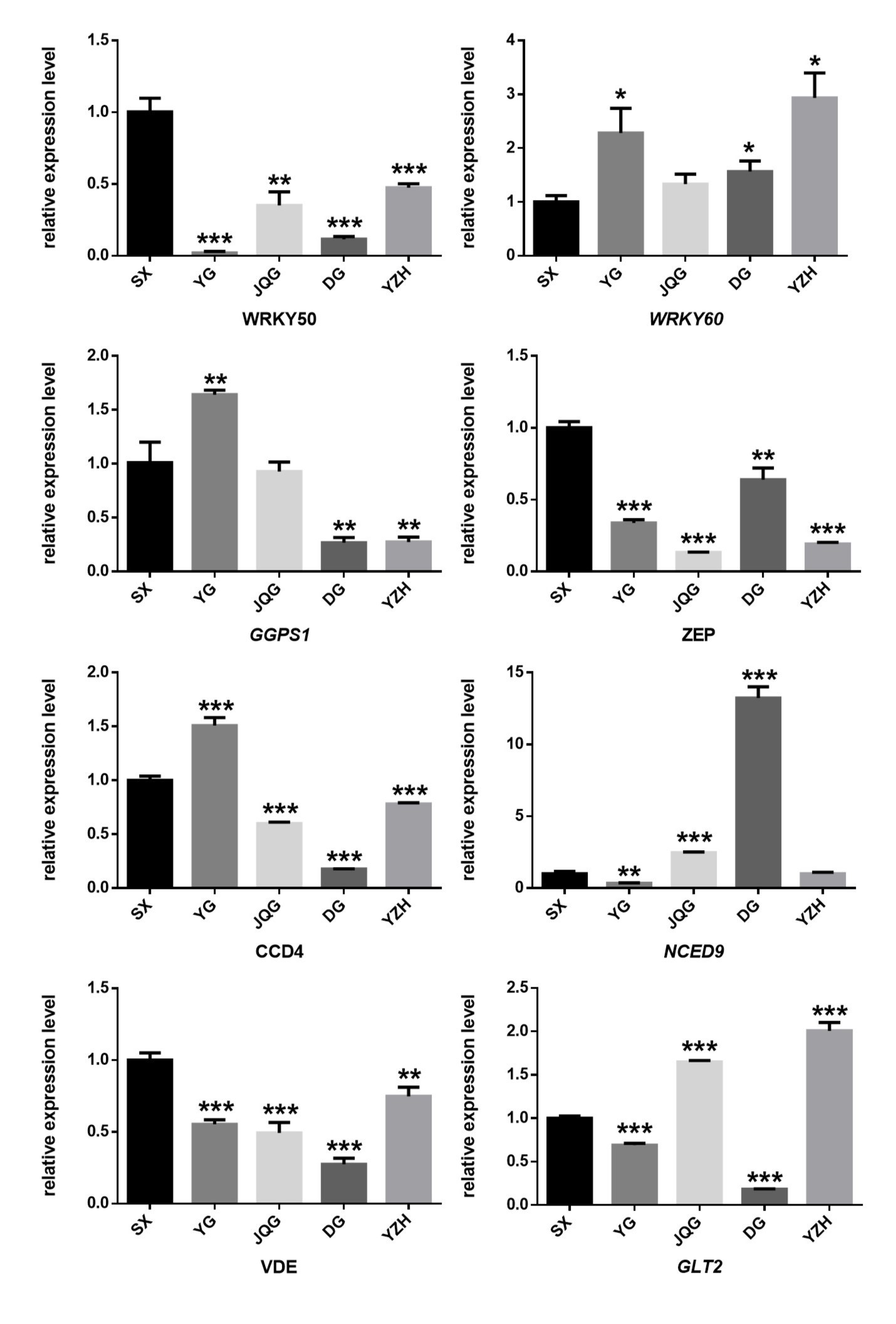
**

**Supplemental Figure 7.** qRT-PCR analysis of candidate genes.

Data were normalized to the untreated control group using the 2^-ΔΔCt method and are presented as mean ± SD (n = 3 biological replicates, ∗*P*<.05,∗∗*P*<.01,∗∗∗*P*<.001). qRT-PCR was performed using SYBR Green on a StepOnePlus Real-Time PCR System (Applied Biosystems).

**
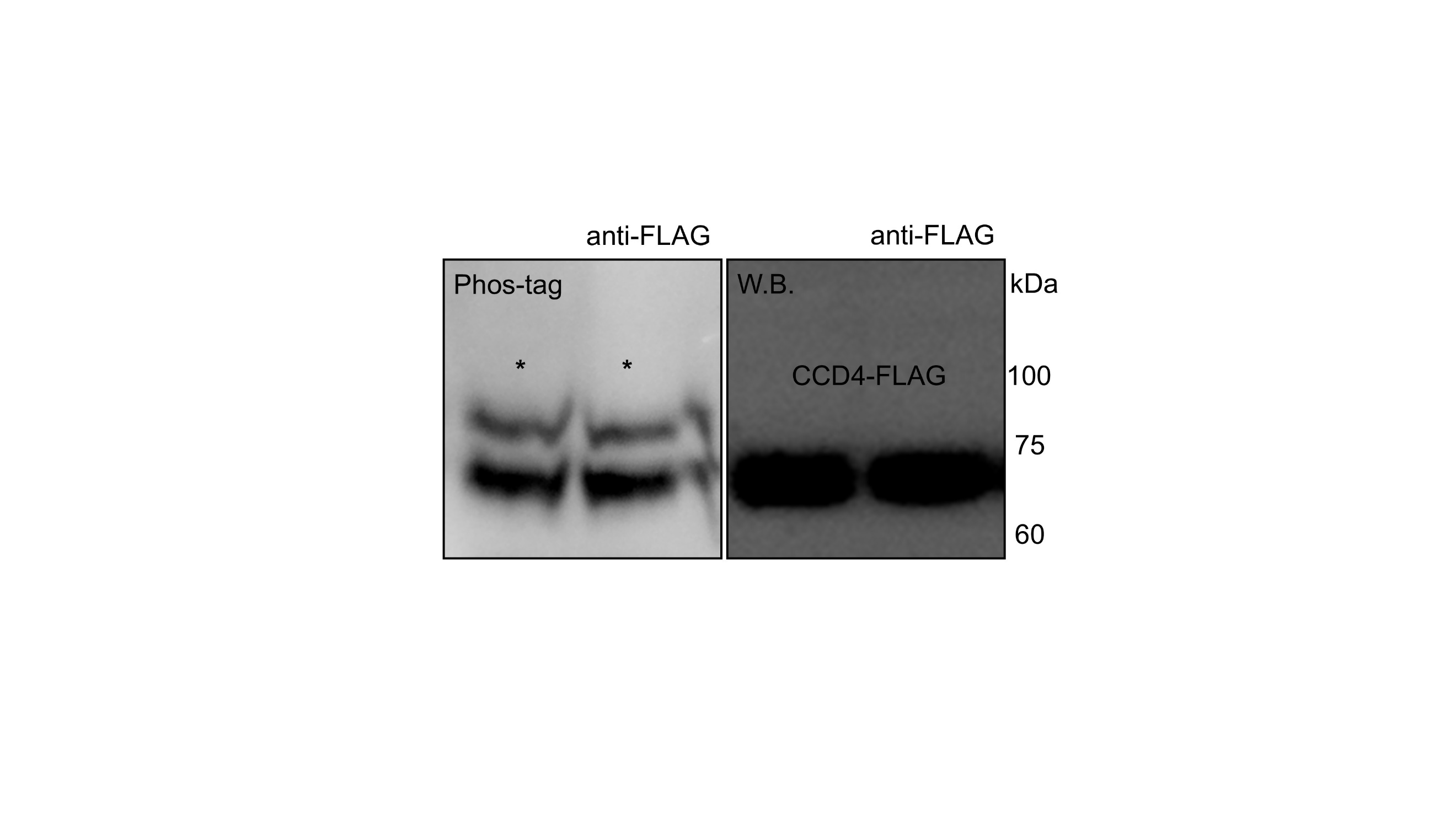
**

**Supplemental Figure 8.** Phosphorylation of CCD4 Protein in vivo.

Representative Western blot showing phosphorylation of CCD4 protein transiently expressed in tobacco leaves.

**Supplementary Table 1.** DAPs related to RNA processing in YG/SX, JQG/SX, DG/SX, and YZH/SX comparisons identified by SWATH-MS-based proteomics.

| **Encoding gene identifier** | **Homolog in Arabidopsis** | **Functional classification** | **Description** |
| --- | --- | --- | --- |
| ofr.gene9764 | AT5G48870 | mRNA splicing | U6 snRNA-associated Sm-like protein LSm5 |
| ofr.gene9687 | AT1G27650 | mRNA splicing | Splicing factor U2af small subunit B-like |
| ofr.gene8814 | AT1G65700 | mRNA splicing | U6 snRNA-associated Sm-like protein LSm8 |
| ofr.gene833 | AT1G02140 | RNA splicing | Protein mago nashi homolog |
| ofr.gene58706 | AT1G02140 | RNA splicing | Protein mago nashi homolog |
| ofr.gene57886 | AT2G33730 | mRNA splicing | DEAD-box ATP-dependent RNA helicase 21-like |
| ofr.gene57663 | AT4G36690 | mRNA splicing | Splicing factor U2af large subunit A-like |
| ofr.gene57538 | AT2G47580 | mRNA splicing | U1 small nuclear ribonucleoprotein A |
| ofr.gene57265 | AT4G30330 | mRNA splicing | Small nuclear ribonucleoprotein E-like |
| ofr.gene57088 | AT2G30260 | mRNA splicing | U2 small nuclear ribonucleoprotein B''-like |
| ofr.gene56603 | AT2G23930 | mRNA splicing | Probable small nuclear ribonucleoprotein G |
| ofr.gene52536 | AT1G20920 | mRNA splicing | DEAD-box ATP-dependent RNA helicase 42-like |
| ofr.gene52386 | AT1G44910 | mRNA splicing | K12821 pre-mRNA-processing factor 40 |
| ofr.gene52248 | AT5G42820 | mRNA splicing | Splicing factor U2af small subunit B |
| ofr.gene47525 | AT2G33730 | RNA splicing | DEAD-box ATP-dependent RNA helicase 21-like |
| ofr.gene46548 | AT5G16780 | mRNA cis splicing | SART-1 family protein DOT2 |
| ofr.gene4595 | AT2G37220 | RNA splicing | 29 kDa ribonucleoprotein A |
| ofr.gene40442 | AT2G13540 | mRNA cis splicing | Nuclear cap-binding protein subunit 1 |
| ofr.gene37424 | AT3G13200 | mRNA splicing | Spliceosome-associated protein CWC15 homolog |
| ofr.gene35260 | AT3G54170 | mRNA splicing | Pre-mRNA-splicing regulator WTAP |
| ofr.gene32062 | AT1G60900 | mRNA processing | Splicing factor U2af large subunit A isoform 1 |
| ofr.gene26306 | AT2G03870 | mRNA splicing | U6 snRNA-associated Sm-like protein LSm7 |
| ofr.gene20896 | AT5G06160 | mRNA splicing | Splicing factor 3A subunit 3-like |

**Supplementary Table 1.** DAPs related to RNA processing in YG/SX, JQG/SX, DG/SX, and YZH/SX comparisons identified by SWATH-MS-based proteomics (continued).

| **Encoding gene identifier** | **Homolog in Arabidopsis** | **Functional classification** | **Description** |
| --- | --- | --- | --- |
| ofr.gene20572 | AT4G30220 | mRNA splicing | Small nuclear ribonucleoprotein F-like |
| ofr.gene19363 | AT4G25280 | RNA splicing | UMP-CMP kinase-like |
| ofr.gene18954 | AT4G30220 | mRNA splicing | Small nuclear ribonucleoprotein F-like |
| ofr.gene15566 | AT5G44200 | mRNA splicing | Nuclear cap-binding protein subunit 2-like |
| ofr.gene13764 | AT4G03120 | mRNA splicing | U1 small nuclear ribonucleoprotein C-like |
| ofr.gene1316 | AT3G44785 | mRNA splicing | K12836 splicing factor U2AF 35 kDa subunit |
| ofr.gene13146 | AT3G54170 | mRNA splicing | Regulation of alternative mRNA splicing |
| ofr.gene1022 | AT3G56790 | mRNA splicing | U6 small nuclear ribonucleoprotein Prp3-like |
| ofr.gene1020 | AT3G55935 | mRNA splicing | U6 small nuclear ribonucleoprotein Prp3 |

**Supplemental Materials and Methods**

**Supplemental Materials and methods**

***O*. *fragrans*** **flower collection and preparation**

Five *O*. *fragrans* varieties, Shenxue (SX), Yingui (YG), Jinqiugui (JQG), Dangui (DG) and YanZhi Hong (YZH), were used. Flowers of YG, JQG, and DG were collected at their full flowering stage from the campus of Nanjing Forestry University. SX and YZH were collected from Jinhua, Zhejiang Province, China. SX and YZH are new varieties bred and generated by the Zhejiang Jinhua Hua‘an Osmanthus Research Institute.

**Extraction and determination of carotenoids**

The cryopreserved lyophilized samples were ground to powders by ball milling (30 Hz, 1 min). In 50 mg of the ground samples, internal standard was added, followed by extraction with a mixture of *n*-hexane/acetone/ethanol (1:1:2, v/v/v) containing 0.01% (g/mL) BHT (2,6-di-tert-butyl-4-methylphenol). The extract was vortexed for 20 min at room temperature. The supernatant was collected by centrifugation. The extraction was repeated once and the supernatant was combined. Saturated NaCl solution was added to the supernatant and mixed well. When the solution is stratified, the upper layer of the solution was collected. After washing by saturated NaC1 solution two more times, the supernatant was dried in a vacuum concentrator. The pellet was saponificated in 10% (v/v) KOH-methanol solution in dark. After saponification, saturated NaC1 aqueous solution and MTBE (methyl tert-butyl ether) were added and mixed well. The upper layer was transferred to a new tube, and was washed by saturated NaC1 aqueous solution twice. The upper layer was dried in a vacuum concentrator. The pellet was resuspended in methanol/methyl tert-butyl ether (3:1, v/v), and stored in a brown injection bottle after passing through a 0.22 μm membrane for LC-MS/MS analysis.

The data collector system consisted of Ultra Performance Liquid Chromatography (UPLC) (ExionLCTMAD) and Tandem Mass Spectrometry (MS/MS) (QTRAP 6500+). YMC C30 (3 μm, 100 mm×2.0 mm i.d.) was used as the liquid chromatography column, and the separation was carried out using methanol/acetonitrile (1:3, v/v) added with 0.01% BHT and 0.1% formic acid (v/v) (A) and methyl tertiary butyl ether added with 0.01% BHT (B) with for separation. Gradient elution program: 100:0 (v/v) for A/B at 0 min, 100:0 (v/v) at 3 min, 30:70 (v/v) at 5 min, 5:95 (v/v) at 9 min, 100:0 (v/v) at 10 min, and 100:0 (v/v) at 11 min. The flow rate was 0.8 mL/min; the column temperature was 28°C; the injection volume was 2 μL. Experiments were conducted with three independent biological replicates. Least-significant difference test (LSD, *P* < 0.05) was used to compare the means. Different letters represent significant differences between groups (*n* = 3, *P* < 0.05).

**Sample preparation for proteomic and phosphoproteomic analysis**

Approximately 1 g of petal of each variety and 3 biological replicates were used. After grinding in liquid nitrogen, petals were suspended in lysis buffer (2.5% (w/v) SDS/100 mM Tris-HCl, 1mM DTT, 10μ/mL PMSF, 10μ/mL cocktail, pH 8.5) and ultrasonicated in ice water for 15 min. Supernatant was collected by centrifugation (16000 g, 20 min). Acetone was added to the supernatant to precipitate the proteins. After centrifugation, the pellets were washed with acetone and air-dried. A solution containing 8 M Urea and 100 mM Tris-HCl (pH 8.0) were added to dissolve the proteins. After centrifugation, dithiothreitol was added to the supernatant at a final concentration of 10 mM, followed by incubation at 37 °C for 1 h. Iodoacetamide was added at a final concentration of 40 mM. Alkylation was carried out at room temperature in dark to seal the sulfhydryl group. Afterward, 100 mM Tris-HCl (pH 8.0) solutions were added to fully dissolve the protein. Protein concentration was determined by the Bradford method. Urea was added to a final concentration less than 2 M. Trypsin (50:1, protein to trypsin) was added and oscillated at 37 °C overnight. The pH value of the solution was adjusted to about 6.0. After centrifugation, the solution was desalted with C18 columns. The desalted peptide solution was dried by a centrifugal concentrator and frozen at -20 °C for mass spectrometry.

**SWATH-MS-based proteomics**

Peptides samples were detected by the TripleTOF 5600+(Sciex) LC/MS system. About 2 µg of peptides were dissolved in MS loading buffer (0.1% formic acid), loaded onto a C18 trap column (5 µm, 5 × 0.3 mm, Agilent Technologies) through auto-sampler and then eluted into a C18 analytical column (75 μm × 150 mm, 3 μm particle size, 100 Å pore size, Eksigent) for separation. The mobile phase consisted of 0.1% (v/v) formic acid, 5% (v/v) DMSO in water (A) and 0.1% (v/v) formic acid, and 5% (v/v) DMSO in acetonitrile (B). Mobile phase A and mobile phase B were used to establish a 60 min gradient, which comprised of: 0 min in 5% B, 40 min of 5-23% B, 5 min of 23-52% B, 1 min of 52-80% B, 80% B for 4 min, 0.1 min of 80–5% B, and a final step in 5% B for 9.9 min. A constant flow rate was set at 300 nL/min. For SWATH scanning, one MS1 scan (ion accumulation time 250 ms, scanning range m/z 350–1500) and 100 MS2 scans with variable windows (ion accumulation time 33 ms, scanning range m/z 100–1800) were included in each cycle. The mass spectrum files were processed by DIA-Umpire to obtain the MS2 spectrum files that can be used for database search. TPP software, Comet, and X!tandem search engines were used to search the database. The retrieved results were used as a spectral library. OpenSWATH algorithm was used for SWATH targeting extraction. A false discovery rate (FDR) of <1% was set as selection criteria. The quantitative intensity information was suSXected to log2 conversion, data filling, and data normalization using the imputation algorithm in Perseus software for difference comparison and t-test analysis. Phosphorylation sites with a ratio of above 5 or below 0.2 (*P* < 0.05) were considered as differentially expressed in this study.

**Data analysis of SWATH-MS-based proteomics**

The correlation of protein quantification was analyzed by corrplot R package to evaluate the reliability of SWATH quantitative proteome data. DAPs were subjected to Gene Ontology (GO) and Kyoto Encyclopedia of Genes and Genomes (KEGG) enrichment analysis by clusterProfiler R package ([Yu et al., 2012](#_ENREF_9)). Pathways of KEGG enrichment analysis was drawn with reference to KEGG mapper ([Kanehisa and Sato, 2020](#_ENREF_2)). Functional classification was based on comparison with homologous proteins in *Arabidopsis thaliana* using the MapMAN BIN system (http://ppdb.tc.cornell.edu/dbsearch/searchacc.aspx). Heatmaps of DAPs between petals of SX and that of other cultivars were drawn by pheatmap R package ([Imran et al., 2018](#_ENREF_1)).

**Data analysis of GWAS**

The sequencing reads of each sample were aligned to the reference genome of *Osmanthus fragrans* ([Yang et al., 2018](#_ENREF_8)) using the BWA software ([Li and Durbin, 2009](#_ENREF_5)). Low-quality alignments were filtered using SAMtools ([Li and Durbin, 2009](#_ENREF_5)), followed by sorting, deduplication (removal of PCR duplicates), and indexing of BAM files using Picard Tools. Variant calling was performed using the GATK software([McKenna et al., 2010](#_ENREF_6)). Subsequently, SNP information from different samples was merged into a single VCF file using bcftools. Further filtering of the VCF file was conducted using Plink software ([Purcell et al., 2007](#_ENREF_7)) based on metrics such as coverage, minor allele frequency (MAF), and linkage disequilibrium (LD). Genome-wide association analysis (GWAS) was performed using Plink software ([Purcell et al., 2007](#_ENREF_7)) to establish associations between SNP loci and flower color. The results of the GWAS were visualized using the "qqman" package in R. SNP loci significantly associated with the traits of interest were identified based on a predefined threshold. Finally, candidate SNP loci were annotated using the SNPef tool to analyze their distribution across genes and infer their potential functional impacts.

**Construction of Weighted Gene Co-Expression Networks and Identification of Modules Associated with Moduel-Traits**

Gene co-expression network analysis was specifically performed on tumour tissues using the R package WGCNA ([Langfelder and Horvath, 2008](#_ENREF_3); [Langfelder and Horvath, 2012](#_ENREF_4)). The soft-threshold of co-expression network clustering was selected according to the FPKM values of all genes in the samples, with R > 0.8 as the norm. Genes with FPKM > 0 in at least nine samples were retained. Different genes were categorized by the dynamic tree-cut method into distinct co-expression modules, and the minimum number of genes in each co-expression module was set to 30. The correlation between different modules and the degree of association (module membership, M) of genes within the modules were calculated. The key modules related to flower color were found for subsequent analysis. Cytoscape V.3.0.0 was used tovisualize the regulatory networks.

**Yeast one-hybrid (Y1H) assay**

Y1H assays using pJG4-5 and pLacZi2u as prey and bait-reporter vectors were performed as described in the Yeast Protocols Handbook (Takara Bio USA, Inc.). Full-length WRKY60 coding sequence was cloned into pJG4-5 vector. The promoter sequence of *ZEP*, *CCD4*, *NCED9*, and *GGPS1* were cloned into pLacZi2u vector. The final prey and bait-reporter vectors were subsequently co-transformed into yeast strain EGY48, and grown on SD plates that lacked uracil and tryptophan and contained X-α-gal.

**Expression and purification of recombinant WRKY60 protein in *Escherichia coli***

The full-length coding sequence of WRKY60 was cloned into pET28a vector. The final vector was transformed into *E. coli* strain BL21 DE3pLysS for protein expression. A 2 ml overnight culture was transformed a 50 ml culture in LB medium containing 50 μg/L Kanamycin. Cultures were grown at 37 °C until OD_600_ reached 0.6. Protein expression was induced by 0.5 mM isopropyl-β-ᴅ-thiogalactopyranoside (IPTG) at 28°C for 6h. Cells were harvested by centrifugation (5000g, 20 min, 4 °C) and resuspended in 5 ml of 1× phosphate-buffered saline (PBS; 140 mM NaCl, 4.3 mM Na_2_HPO_4_, 2.7 mM KCl, 1.47 mM K_2_HPO_4_, pH 7.3) containing 5 mM sodium ascorbate. Cells were lysed by sonification on ice four times for 30 s at 10% of the maximal power. Cell debris was removed by centrifugation (5000g, 30 min, 4 °C). His-tagged WRKY60 protein was purified using His-tag purification resin and an affinity chromatography column (Beyotime).

**Microscale thermophoresis (MST)**

Microscale thermophoresis (MST) was employed to assess the affinity between WRKY60 protein and the promoter regions of downstream genes, including *ZEP*, *CCD4*, *NCED9*, and *GGPS1*. MST leverages the differential responses of distinct molecules to a temperature gradient. The concentration of red-labeled WRKY60 was kept constant, while the concentration of the unlabeled binding partner was systematically adjusted. The unlabeled binding partner was titrated in 1:1 dilution, commencing at a concentration of 250 µM.

For MST analysis, the samples were diluted in an MST optimized buffer (PBS). Subsequently, equal volumes of WRKY60 proteins (10 µL) were combined with varied DNA concentrations and thoroughly mixed. The resulting mixtures were loaded into capillaries provided by Nano Temper Technologies. Measurements were conducted following a 10-minute equilibration at room temperature. The measurements were carried out using auto LED power and medium MST power settings.

**Luciferase assays**

The promoters of *GGPS1*, *ZEP*, *CCD4*, *NCED9*, *WRKY60*(SX), *WRKY60*(YG), *WRKY60*(JQG), *WRKY60*(DG), *WRKY60*(YZH) were amplified and cloned into the vector pGreen-0800. Full-length WRKY60 coding sequence was cloned into pGreen-62-SK vector. The Pro: LUC-35S: REN reporter constructs were transformed into A. tumefaciens strain GV3101 and injected together with the control vector or the pGreenII-62-SK::WRKY60 effector into leaves of *N. benthamiana*. The leaves were sampled at 40 h post-transfection. Images of the LUC signal were obtained using a Tanon 5200 Multi Chemiluminescent Imaging System. The LUC and REN activities were determined using a Dual Luciferase Reporter Gene Assay Kit (Vazyme, China), with the LUC/REN ratio representing the relative activity of the promoter.

**Phosphorylation of CCD4 in Vivo Assays**

Constructs to investigate the phosphorylation of CCD4 was produced in pUC19-UBQ10. Agrobacterium tumefasciens (strain GV3101) cultures were transformed with the plasmids by using the freeze-thaw method. They were then grown in liquid Luria Broth to late exponential phase, and cells were harvested by centrifugation and resuspended in 10 mM MES acid-KOH, pH 5.6, containing 10 mM MgCl2 and 150 mM acetosyringone to an OD600 of 1. These cells were mixed with an equal volume of Agrobacterium C58C1 (pCH32 35S: p19) expressing the silencing suppressor p19 of tomato bushy stunt virus so that the final OD600 of the Agrobacterium solution was ∼1. Bacteria were incubated for 3 h at room temperature and then injected into young fully expanded leaves of 4-week-old *N. benthamiana* plants. Total protein was extracted from tobacco leaves 2–3 days after infiltration using 1% SDS extraction buffer. Proteins were then fractionated by SDS–PAGE and Mn^2+^-phos-tag-PAGE (50 μM phos-tag and 100 μM Mn^2+^).

**Supplemental References**

**Imran, Q.M., Hussain, A., Lee, S.-U., Mun, B.-G., Falak, N., Loake, G.J., and Yun, B.-W.** (2018). Transcriptome profile of NO-induced Arabidopsis transcription factor genes suggests their putative regulatory role in multiple biological processes. Sci Rep **8**:771-771. 10.1038/s41598-017-18850-5.

**Kanehisa, M., and Sato, Y.** (2020). KEGG Mapper for inferring cellular functions from protein sequences. Protein Sci **29**:28-35. 10.1002/pro.3711.

**Langfelder, P., and Horvath, S.** (2008). WGCNA: an R package for weighted correlation network analysis. BMC Bioinformatics **9**:559. 10.1186/1471-2105-9-559.

**Langfelder, P., and Horvath, S.** (2012). Fast R Functions for Robust Correlations and Hierarchical Clustering. J Stat Softw **46**.

**Li, H., and Durbin, R.** (2009). Fast and accurate short read alignment with Burrows–Wheeler transform. Bioinformatics **25**:1754-1760. 10.1093/bioinformatics/btp324 %J Bioinformatics.

**McKenna, A., Hanna, M., Banks, E., Sivachenko, A., Cibulskis, K., Kernytsky, A., Garimella, K., Altshuler, D., Gabriel, S., Daly, M., et al.** (2010). The Genome Analysis Toolkit: A MapReduce framework for analyzing next-generation DNA sequencing data. GENOME RESEARCH **20**:1297-1303. 10.1101/gr.107524.110.

**Purcell, S., Neale, B., Todd-Brown, K., Thomas, L., Ferreira, M.A.R., Bender, D., Maller, J., Sklar, P., de Bakker, P.I.W., Daly, M.J., et al.** (2007). PLINK: A Tool Set for Whole-Genome Association and Population-Based Linkage Analyses. The American Journal of Human Genetics **81**:559-575. <https://doi.org/10.1086/519795>.

**Yang, X., Yue, Y., Li, H., Ding, W., Chen, G., Shi, T., Chen, J., Park, M.S., Chen, F., and Wang, L.** (2018). The chromosome-level quality genome provides insights into the evolution of the biosynthesis genes for aroma compounds of Osmanthus fragrans. Horticulture Research **5**:72. 10.1038/s41438-018-0108-0.

**Yu, G., Wang, L.G., Han, Y., and He, Q.Y.** (2012). clusterProfiler: an R package for comparing biological themes among gene clusters. Omics **16**:284-287. 10.1089/omi.2011.0118.
